# Patient preferences and performance bias in a weight loss trial with a usual care arm^☆^^☆☆^

**DOI:** 10.1016/j.pec.2014.01.003

**Published:** 2014-05

**Authors:** Jim McCambridge, Annik Sorhaindo, Alan Quirk, Kiran Nanchahal

**Affiliations:** a Department of Social & Environmental Health Research, London School of Hygiene & Tropical Medicine, London WC1H 9SH, UK; b Royal College of Psychiatrists’ Centre for Quality Improvement, London E1 8AA, UK

**Keywords:** Performance bias, Weight loss, Patient preferences, Patient counseling, Behavior change, Research participation, Disappointment

## Abstract

**Objectives:**

This qualitative study examines performance bias, i.e. unintended differences between groups, in the context of a weight loss trial in which a novel patient counseling program was compared to usual care in general practice.

**Methods:**

14/381 consecutive interviewees (6 intervention group, 8 control group) within the CAMWEL (Camden Weight Loss) effectiveness trial process study were asked about their engagement with various features of the research study and a thematic content analysis undertaken.

**Results:**

Decisions to participate were interwoven with decisions to change behavior, to the extent that for many participants the two were synonymous. The intervention group were satisfied with their allocation. The control group spoke of their disappointment at having been offered usual care when they had taken part in the trial to access new forms of help. Reactions to disappointment involved both movements toward and away from behavior change.

**Conclusion:**

There is a prima facie case that reactions to disappointment may introduce bias, as they lead the randomized groups to differ in ways other than the intended experimental contrast.

**Practice implications:**

In-depth qualitative studies nested within trials are needed to understand better the processes through which bias may be introduced.

## 1 Introduction

Performance bias refers to the conduct of a trial inadvertently introducing differences between randomized groups other than the intervention(s) being evaluated. Such departures from intended study design may compromise study aims by undermining capacity to make valid inferences about intervention effects. In healthcare contexts, staff provision of differential care when there is a lack of blinding about randomization status constitutes a classic example of this phenomenon. Indeed differential care has been included within the definition offered by the Cochrane Collaboration as “systematic differences between groups in the care that is provided, or in exposure to factors other than the interventions of interest” [1]. Whilst considered in the context of systematic reviews [e.g. [2]] and related research methods texts, it is not obvious that this construct has itself been subjected to empirical research scrutiny.

Randomization is a somewhat unusual process as chance does not overtly govern many decisions in people's lives, and this may provoke apprehension in advance or result in disappointment for some trial participants [3]. Randomization is important in health sciences and is widely used for good reasons, though paradoxically its direct effects are rarely measured [4]. Placebo control conditions are used in trials to manage the possible effects of disappointment, as well as to take account of the placebo effect itself [5]. Due to concerns such as these, patient preference designs have been developed to avoid randomizing participants with strong allocation preferences to study conditions that would be disappointing [6]. Some systematic reviews have identified capacity of preferences to impact on trial outcomes [7] whereas others have not [8].

Zelen designs have also been developed for situations where seeking consent to be randomized may be problematic [9]. Systematic reviews provide evidence of the use of Zelen and patient preference designs in many areas [8,10], which might suggest that the underlying problems associated with disappointment, and their implications, are well understood.

There have been valuable studies of public understanding of various aspects of randomization [11,12]. Qualitative studies have identified preferences to be potentially complex and dynamic, as well as being amenable to dedicated interventions [13]. How information about randomization is presented in seeking informed consent has received scrutiny [14] and dedicated interventions have successfully enhanced informed consent and recruitment to trials [15]. There are also qualitative studies investigating whether and how trial participants react to being randomized [16], though most such studies have been undertaken in clinical contexts where contextual effects may be pronounced, such as neo-natal intensive care units [17].

Cook and Campbell [3] have suggested possible responses to disappointment, ranging from control group participants trying harder by accessing interventions outside trials (termed “compensatory rivalry”) to participants giving up as a result of disappointment (“resentful demoralization”). Without control of such reactions, trials may be vulnerable to performance bias (1). One leading trialist [18] has gone as far as to suggest that “the next substantive milestone in the history of efforts to create unbiased comparison groups may be erected when someone solves the interesting methodological conundrum presented by biases resulting from patient preferences”.

Randomized controlled trials, like other research studies, involve interactions between participants and researchers. Patient preferences may have implications for the actual conduct of these studies, although trial design seeks to preclude this possibility, along with any impact on trial outcomes. This preliminary investigation explores how patient preferences may be associated with performance bias in one trial by examining reasons for participation and participant engagement with the research study. In so doing, it seeks to offer a participant-centered view of what it is like to become involved in a trial, in order to better appreciate the potential for biases that stem from research participation itself, which may not be well understood [19].

## 2 Methods

Case studies are investigations which pay particular attention to the contexts in which data are produced [20]. This is a case study particularly concerned with patient preferences and possible performance bias in the CAMWEL (Camden Weight Loss) trial evaluating a patient counseling intervention delivered in primary care to help people lose weight in comparison with usual care [21]. We thus emphasize the patient counseling evaluation study context and the intrinsically unblinded nature of this contrast, where usual care was familiar to participants. Lifestyle interventions in the primary care setting are widely recognized as being important for public health purposes, so such studies must be as rigorous as possible [22]. A brief trial description is provided below, with further details available elsewhere [21].

The CAMWEL trial evaluated the effectiveness of a structured one-to-one support program delivered in primary care over a 12 month period by trained advisors for overweight or obese people who wished to lose weight among residents of Camden, an ethnically diverse inner London borough with a mix of areas of relative affluence and deprivation. The trial participants were 381 adults with body mass index (BMI) ≥ 25 kg/m^2^ recruited in 23/39 National Health Service (NHS) Camden general practices between July 2009 and January 2010 [21]. The trial was pragmatic in nature so as to be generalizable across the UK NHS, with as few exclusion criteria as possible [21]. Brief telephone screening was followed by a face-to-face appointment with a researcher for informed consent, baseline questionnaire completion and anthropometric measures. Participants were randomly allocated to the patient counseling program being evaluated or to usual care, which is general practitioner management, potentially involving prescription of weight loss drugs, referral to dieticians or for weight loss surgery [21].

Process studies are recommended within trials to confirm that the study is being implemented as intended and to explore intervention delivery issues, contextual factors and possible mechanisms linking processes to outcomes [23]. The CAMWEL process study collected semi-structured interview data from 34 (17 in each arm) of the 381 trial participants who were purposively selected to be diverse in gender, age, education and baseline weight. Participants provided separate consent to take part in the process study. The trial was approved by the London School of Hygiene & Tropical Medicine (LSHTM) Ethics Committee, the Camden and Islington Community Research Ethics Committee (REC Reference number 09/H0722/22), and the North Central London Research Consortium.

The purpose of this study is to explore to what extent participants’ reactions to being randomized, in the context of their decision to take part in the trial, inform understanding of the construct of performance bias. During the first process study interview, undertaken usually in the weeks following communication of the outcome of randomization by telephone, we investigated what impact the conduct of the trial had on 14 consecutive process study participants (8 control group, 6 intervention group). The data presented here in this preliminary investigation are participants’ responses to a small number of dedicated questions about the process of study participation in a 30-min interview, primarily undertaken for other purposes. Participants were invited to recall how they found out about the study and were asked for example, “what was your main reason for taking part” and “what were your hopes for taking part in the study”. This invitation extended chronologically to all their early contacts up to and including randomization with invitations such as “If you could just think back to the screening visit…what do you remember”. Participants thus recounted their experiences and answered questions such as “after you came out of the screening visit, did you think anything differently about your weight?” and after communication of allocation, “how did you feel about that?” The data were not collected in an inductive manner, with each interview being informed by the previous interviews; rather, the same topic guide was used for all interviewees. All interviews were conducted by the second author, digitally recorded and later transcribed. Most took place in the GP practice where the participant had been assessed, with some also on the premises of LSHTM or via telephone, at the convenience of the participant.

Data relating to patient preferences (mostly made up of the responses to the dedicated questions) were retrieved and examined independently by JM and AS. Each drafted a coding frame, after which a consensus meeting was held to agree on the final set of codes, which the first author applied to the dataset using word processing software. A thematic content analysis of these data was undertaken, which focused on latent rather than manifest patterns of meaning [24]. The coding and analysis is best described as primarily deductive in that it was led by author JM who looked for concepts previously described in relevant literature. That noted, both analysts were open to types of research participation effects that had not previously been identified, as is reflected in the Results below. With assistance at the writing-up stage from author AQ, an experienced qualitative analyst, themes that were not substantial enough were excluded from the report, i.e. where the data were insufficient to reach theoretical saturation. Data from individual participants are presented by participant number, with the group to which they belonged indicated by Intervention Group [IG] or Control Group [CG] as appropriate. To shorten quotes and make them easier to read, parts of the utterance have been omitted. These are represented by bracketed ellipsis: […].

## 3 Results

We present data on reasons for participation, prior to examining the reactions of the control group and the intervention group to their allocation. The concepts of ‘conditional’ or ‘weak’ altruism have been developed to describe reasons for participation that benefit both the individual concerned and wider society [25,26]. There was some evidence for this in our sample:

I got this circular which said they were looking for volunteers and I thought ‘well it might be helpful to me as well as helpful to someone else’. (Participant 3 [IG])

However, the invitation to participate in the trial was much more commonly perceived as a “helping hand” and an opportunity to access new forms of help:

Actually, it was more of a helping hand. You know what I mean? It was someone come and help me. So that's why I replied almost straight away, like, you know, so when I got home that night I was ‘let's have a go at this, see what this brings’ (Participant 14 [IG])

I was thinking about doing something about [losing weight] but I didn’t know how. (Participant 8 [IG])

Well I was hopeful, maybe, there would be something new, if it would help me lose weight, you know, or something better than what I’ve tried before because the things I’ve done before don’t work very well. (Participant 2 [CG])

Others perceived it as an opportunity to learn how to manage their weight better:

I thought that with this studies thing that maybe it would help me to understand how best maybe for me to go with my weight management. (Participant 4 [IG]))

What it made me think was, you know, that my GP was obviously taking my concerns quite seriously and he's trying to help me (Participant 12 [CG])

This study shows the obvious potential for conflict between seeking new forms of help as a reason for participation and the possibility of being allocated to a familiar usual care control group in which no new help is provided. This situation contrasts with other trials in which people may prefer allocation to standard practice as compared to innovations that are untested [27].

Participants generally appreciated the uncertainty involved in random allocation, if not the technical details, though the possibility that they might not get the novel intervention was not always prominent in the accounts provided. One participant spoke of her disappointment at having not been put in the “favored group”:

I suppose truthfully, [I was] a bit disappointed, but not for long because it's a research project. I just would have liked to have been in what I then considered the favored group! Of course because, you know, I think that that will work better for people and I presume that is the hypothesis. (Participant 10 [CG])

The strength of disappointment expressed by control participants varied widely. One spoke of being “a bit miffed” (a colloquial term for strong disappointment):

The only thing I was disappointed about was because I did not actually get the [arm of the] trial I wanted **[…]** That's where I was, kind of, really a bit miffed because I really, really wanted to do that program ‘cos I thought that program was good for me, but it did not happen. (Participant 13 [CG])

For another, allocation to the control group had left her feeling “totally disgusted” because it had meant that “nobody wants to help me”:

The truth? Totally disgusted. ‘Cos I thought I was going to get some help and nobody wants to help me. You know, and I have put on weight since the last time I came, I know I have. I feel it in my body and the scales say I have. And I just want someone to help me. (Participant 6 [CG])

This appears a good example of resentful demoralization. Another control group participant drew on pre-existing knowledge of science to rationalize acceptance of their allocation:

Well I understand science so I know there has to be a sample group that is not participating, as a control group. So I knew that I could possibly get into the control group which I did, so I just accepted that. **[…]** I was kind of disappointed because I was hoping for something different, instead I do not have anything at all really. (Participant 2 [CG])

Awareness of the trial context aroused curiosity in one participant:

I’ve gone to see my GP, that's sort of prompted me to ask him, I was just more curious **[…]** The point was, I kind of went to see him out of, purely **[…]** knowing there was a trial in place so I knew there were two programs, so it sort of spurred me on to, sort of, see what they would offer. (Participant 5 [CG])

Allocation to the control group was perceived by another participant to have not given them “more of an incentive” to lose weight:

You think of something like this as giving you more of an incentive except that so far it hasn’t **[…]**. It's like it's doing some kind of an experiment, which we do not really know what it is. (Participant 1 [CG])

There was some evidence for compensatory rivalry [3] in our sample, but of a type not previously described in the literature:

Well, I do not think I have any particular hopes actually since I was assigned to the control group, as it were. I think I’ve decided to take my own control again. (Participant 10 [CG])

What is novel here is the comment “I think I’ve decided to take my own control”, which implies a decision on the participant's part to change their behavior themselves, rather than necessarily seeking alternative interventions outside the trial. Such a response may be specific to trials where change in behavior can be achieved without the aid of external resources.

Seven of the 8 control group participants expressed disappointment, whereas all participants in the intervention group were satisfied with their allocation. In some cases, they were simply pleased to be receiving some additional support, as usual care was seen as insufficient.

I think I was more pleased because I know that GPs are extremely busy, they hardly have time to talk to you, or hear what you’re saying. (Participant 8 [IG])

I think I’d be faintly disappointed if I was, you know ‘you’re the placebo control group, you just carry on!’(Participant 11 [IG])

Others hoped to be provided with “expert help”.

I just feel that, in the intervention group, maybe there’ll be some kind of other help rather than just the GP. I’m not saying that going to the GP was a waste of time ‘cos it wasn’t ‘cos I then went to their nutritionist and she went through my diet sheet and stuff with me. I’m just happy that I am in the intervention group ‘cos I’m hoping, now, that there’ll be some kind of expert help for me. (Participant 4, IG)

## 4 Discussion and conclusion

### 4.1 Discussion

This study explored how patient preferences may be associated with performance bias in CAMWEL by examining reasons for participation which involve preferences and how participants react to disappointment when their preferences are thwarted. Participants were disappointed at being randomized to usual care because preference for the intervention arm was the principal reason for participation. While they had not been apprehensive about the use of chance as an allocation mechanism, their reactions to being randomized to usual care ranged from being “spurred on” to explore usual care (Participant 5) and deciding to assert “own control” (Participant 10) to being “totally disgusted” (Participant 6) at not being offered additional help. The reactions captured here include those speculated about by Cook and Campbell (3) more than 30 years ago. Whilst there is a longstanding literature on reasons for participation in research, there is not a body of work on how reasons for participation may impact on trial outcomes. Patient preferences may impact on trial outcomes [7,8], and this study contributes a new understanding of some mechanisms by which this may occur. These issues are not specific to patient counseling or behavioral intervention trials [28].

Historically, altruism has been seen as the key motivation for all forms of research participation [25,26], so it is striking how small a role altruism seemed to have played in people's decisions to participate in this trial. The specific circumstances of evaluating new methods of helping people change well established behaviors, particularly where there have been past attempts to change, may militate against altruism. Where conditional altruism was reported, altruistic reasons appeared much weaker than the primary motivation of help-seeking. Attempts made by some participants to be good subjects were accompanied by uncertainties about their roles and what was expected of them.

The reasons for participation articulated here appear *prima facie* to have clear implications for how participants respond to their allocated study condition. The lack of fit between reasons for participation (to access to new forms of help) and the content of the control condition (usual care) explains the thwarted preference and disappointment in this trial, but not participants’ *reactions* to their disappointment. This is important in relation to possible performance bias, which is concerned with unintended aspects of the conduct of the study. The origins of the reactions captured here lie implicitly within the design of the trial itself, where there is potential for conflict between reasons for participation which involve preferences and the outcome of randomization.

It is a moot point whether performance bias is the most appropriate conceptualization of this problem, yet these reactions do deserve to be recognized as a distinct source of bias. This is because they lead the randomized groups to differ in ways other than the intended experimental contrast. It may be that a conceptualization is needed that distinguishes unintended differences between groups in how participants are *treated* in the conduct of the trial (performance bias) from systematically different *reactions* between randomized groups to identical trial procedures. A different definition of performance bias that is not restricted to how participants are treated by the study may be useful, and more fine grained attention to how participants react to what they are asked to do in research, and how this may impact on study outcomes, is needed.

The possible direction and magnitude of bias is important to consider. In this trial, there was some evidence of small differences in outcomes, though not in the primary outcome of weight loss [21]. Inferences about effectiveness in studies which find differences between groups must take account of the possibility that the types of reactions described here may be responsible for some of these differences if it seems possible or likely that disappointment may be involved. Compensatory rivalry responses to the outcome of randomization may attenuate differences between groups and resentful demoralization may exaggerate them, so the former are particularly worth considering where there are null findings. In this study, we saw evidence of both, and there is thus no strong evidence that trial findings are systematically biased in this instance.

Usual care or standard practice is a very common control condition. Indeed, it is the standard against which innovations should be assessed for ethical as well as methodological reasons, hence its incorporation into the Helsinki Declaration [29]. In this trial, and in many other evaluations of patient counseling in which comparisons are made with usual care, blinding the participant to their group allocation was impossible because usual care was familiar and the novel intervention being evaluated obviously distinct. Although unlikely for chronic conditions as seen here, blinding allocation to usual care remains possible in circumstances where usual care has not previously been received.

There are clear limitations to the present study where our findings are based on relatively brief enquiries nested within interviews with a small number of trial participants about psychological and behavioral processes which are both long running and complex. This study should thus be considered as hypothesis generating for methodological investigations, revealing possible mechanisms for the introduction of bias. We draw attention to the need to better conceptualize and study how reasons for participation may imply preferences in trials, and possible mechanisms for the introduction of bias specifically induced by disappointment due to thwarted allocation preferences. More generally, we should address how motivational and other factors associated with research participation itself, including specific roles or activities required of participants, may bias study outcomes and thus undermine study aims, both for trials and other study designs [30–34].

### 4.2 Conclusions

Efforts to access a novel counseling intervention within a trial, when there have been prior attempts at weight loss, resulted in satisfaction if successful, and disappointment if unsuccessful. There is a prima facie case that reactions to disappointment may introduce bias, as they lead the randomized groups to differ in ways other than the intended experimental contrast. There is a need to better identify disjunctures between reasons for participation and the content of allocated study conditions in trials. It is possible that there is widespread bias in trials where there are such disjunctures. There is a clear need to discover where this overlooked threat to valid inference in trials is most acute and also whether our understanding of performance bias provides the best guide to empirical study of these issues.

### 4.3 Practice implications

This study has implications for trialists and not directly for clinical practice. There is a widespread tendency within the research community to view research procedures as inert [35] and not influencing participant cognitions, emotions and behavior. This was clearly not true for the participants in this trial. Invitations to participate in trials and subsequent study requirements may interact in complex ways with people's ongoing struggles to lose weight. Having such a dynamic conceptualization suggests the need for in-depth qualitative longitudinal investigations nested within trials of participant experiences. This study has obvious implications for the design of trials with usual care control conditions which are unblinded for participants, where participants prefer to avoid being allocated to these study conditions. Preferences should be assessed and alterations to conventional trial designs, such as patient preference designs, should be considered where strong preferences appear likely.

We confirm all patient/personal identifiers have been removed or disguised so the patient/person(s) described are not identifiable and cannot be identified through the details of the story.

## Conflict of interests statement

The authors declare that there is no conflict of interest.

## Contributors

JM had the idea for the study, led the data analyses and wrote the first draft of the report. AS undertook the interviews and participated in analysis of the resulting data. AQ assisted in conceptual work and data presentation. KN was the Principal Investigator for the CAMWEL trial and is the guarantor for this study. All authors participated in discussions about the design of this study, contributed to revisions of the report and approved the submission of the final report.

## Funding

The CAMWEL trial was funded by the Camden Primary Care Trust (NHS Camden). JM is supported by a Wellcome Trust Research Career Development Fellowship in Basic Biomedical Science (WT086516MA). The sponsor and funder had no role in the decision to publish nor in the writing of this paper.
